# Fecal microbiota transplantation treatment of autoimmune-mediated type 1 diabetes: A systematic review

**DOI:** 10.3389/fcimb.2022.1075201

**Published:** 2022-12-01

**Authors:** Shuo Zhang, Feiying Deng, Jingxian Chen, Fengwu Chen, Zezhen Wu, Liping Li, Kaijian Hou

**Affiliations:** ^1^ Shantou University Medical College, Shantou, China; ^2^ Department of Endocrine and Metabolic Diseases, The First Affiliated Hospital of Shantou University Medical College, Shantou, China; ^3^ Department of Endocrine and Metabolic Diseases, Longhu People’s Hospital, Shantou, China; ^4^ School of Public Health, Shantou University, Shantou, China

**Keywords:** Autoimmune-mediated type 1 diabetes, gut microbiota, fecal microbiota transplantation, chronic inflammatory state, insulin resistance

## Abstract

There is a strong link between fecal microbiota and the development of type 1 diabetes. As an emerging therapeutic modality, fecal microbiota transplantation has been shown to be safe and effective in the treatment of many intestinal and extraintestinal diseases. Various studies have found that fecal microbiota transplantation can treat diseases by correcting patients’ immune disorders. Besides, many studies have found that fecal microbiota transplantation can improve glycemic control and insulin resistance in diabetic patients. Therefore, this paper reviews the mechanism of action of fecal microbiota transplantation on autoimmune-mediated T1DM and the current research progress, feasibility, and issues that need to be addressed in the future development of fecal microbiota transplantation in the treatment of autoimmune-mediated T1DM.

## Introduction

### Current status of type 1 diabetes mellitus

Diabetes mellitus is a group of diseases characterized by chronic hyperglycemia caused by multiple etiologies, which can cause damage to multiple systems of the body and bring about various acute and chronic complications (Hou et al., 2021). The most common types of diabetes mellitus are type 1 diabetes mellitus (T1DM), type 2 diabetes mellitus (T2DM), other specific diabetes mellitus, and gestational diabetes, and they can all be summarized as a chronic inflammatory state (American Diabetes Association Professional Practice C. 2, 2022). T1DM is also known as a type of insulin-dependent diabetes mellitus, which mostly starts in adolescence or childhood. According to the International Diabetes Federation (IDF), 536.6 million adults aged 20–79 years in 215 countries and territories have been diagnosed as diabetes patients by 2021, and 1.2 million children and adolescents under 20 years of age have been diagnosed with type 1 diabetes, and there will probably be 149,500 children and adolescents with type 1 diabetes by 2045 (Sun et al., 2022). The systemic neurological and vascular dysfunction caused by T1DM can affect the cardiac vessels, nerves, eyes, and kidneys (Nicholson et al., 2012). Its complications and mortality account for approximately 5-10% of the global diabetes financial burden (Mobasseri et al., 2020). The current incidence of T1DM is increasing at 3% to 5% per year, which will cause a severe social and economic burden (Wang et al., 2017), and global diabetes-related health expenditures are estimated to reach $1,054 billion by 2045 (Sun et al., 2022). At present, the dominant treatment for T1DM is still to reduce blood glucose by injecting insulin, but this is only a symptomatic treatment.

### T1DM etiology and influencing factors

At present, T1DM is composed of two subtypes, including the autoimmune type (T1A) and the non-autoimmune type (also known as idiopathic type 1 diabetes mellitus (T1B), of which T1A accounts for the majority (American Diabetes Association Professional Practice C. 2, 2022). On the one hand, T1DM patients are in an autoimmune-mediated inflammatory state, producing a variety of inflammatory factors, such as TNF- α, IL-1, and IL-6 (Vatanen et al., 2016); on the other hand, innate and adaptive immunity mediates the production of autoantibodies, such as glutamic acid decarboxylase antibodies and zinc transporter 8 antibodies, both of which will lead to the damage of β cell function and insulin secretion (Abdellatif and Sarvetnick, 2019). Genetic factors, harmful factors in the environment, bacteria, fungi, and viral infections all might make pancreatic β cells exhausted and eventually fail due to a secondary autoimmune destruction (Rewers and Ludvigsson, 2016). In the population, different seasons and geographical locations (Samuel et al., 2008; Kimura et al., 2013), changes in diet and delivery methods, and the use of antibiotics will also affect the incidence of T1DM (Krauss, 2004). For example, in early life, exposure to environmental chemicals and air pollution can affect the development of the immune system, and the function and survival of β cells leading to an increase in the incidence rate of T1DM (Malmqvist et al., 2015). The different components of drinking water in different regions will also affect the incidence rate of T1DM, it has been found that the content of some metal elements, barium, and nickel, is negatively correlated with the incidence rate of T1DM (Chafe et al., 2018). Viruses mainly include enteroviruses and Coxsackie B viruses. Human infection with related viruses can induce pancreatitis or produce substances similar to islet autoantigens, thus activating the immune system and leading to or accelerating the progress of T1DM (Stankov et al., 2013). Among genetic factors, more than 50 T1DM-susceptible genes have been found through family linkage analysis and genome-wide association studies. Different genes will lead to variable effects on T1DM susceptibility, among which HLA-DR and DQ genes are the most closely related, accounting for 40% to 50% of the pathogenic risk factors (Ziegler and Nepom, 2010). Although HLA-DR risk alleles increase the susceptibility of high-risk children to T1DM, only 5% or fewer genes will lead to the development of T1DM (Krischer et al., 2019). Nongenetic modification factors such as diet, gut microbiota, pressure, and chemical and environmental factors play an essential role in the occurrence and development of T1DM (Mullaney et al., 2018). Therefore, the limited residual of T1DM patients is retained through nongenetic factors β Cell function is crucial for the quality of life and prognosis of patients (Wang and Jia, 2016). More and more studies have found that gut microbiota plays a crucial role in the occurrence and disease progression of T1DM. The transplantation treatment around gut microbiota can effectively improve the gut microbiota imbalance in patients, which holds promise for improving glycemic control and insulin resistance in patients with T1DM (Steffes et al., 2003).

## T1DM and gut microbiota

More and more studies show that gut microbiota (GM) is closely related to the occurrence and development of T1DM. The pathophysiological changes of T1DM are related to the changes in GM. The GM can affect the progress of T1DM in many aspects (de Goffau et al., 2014; Davis-Richardson and Triplett, 2015; de Groot et al., 2017). “Gut microbiota” refers to more than 1014 kinds of bacteria, fungi, viruses, and others residing in the gastrointestinal tract and performing various functions in the gastrointestinal tract. The “microbiota” is considered the genome of the entire microbiota (Abdellatif and Sarvetnick, 2019). The intestinal tract of humans is composed of about 100 trillion bacterial cells, 10 times the total number of human cells. The microbiota weighs 1.5 kg and has more than 3.3 million genes, which is 150 times the human gene (Pitocco et al., 2020), showing that GM can play an essential role in our body. GM is mainly divided into four types at the phyla level. The first is Firmicutes (gram-positive), which constitutes 60-80% of the microbiota, including more than 200 genera (the most important ones are Rumen coccus, Clostridium, and Lactobacillus); the second is Bacteroides (gram-negative, including Bacteroides, Prevotella, and Trichoderma), accounting for 20-30% of the microbiota; the next is actinomycetes (gram-positive), accounting for about 10% of the microbiota (mainly Bifidobacterium); and finally, Proteus, such as Escherichia coli and Enterobacteriaceae (Hou et al., 2022). Therefore, we can see the close relationship between gut microbiota and T1DM, and the feasibility of fecal microbiota transplantation with gut microbiota as the therapeutic target.

### Insulin resistance is influenced by gut microbiota

The insulin resistance is the leading risk factor and feature of T2DM. Although the main cause of T1DM is the absolute lack of insulin secretion, most patients have insulin resistance at the same time, and this feature runs through the beginning of the disease and the subsequent insulin treatment process. Some patients have a trend of increasing insulin demand in the subsequent clinical treatment, which reflects the rising insulin resistance index (Homa IR) (Pedersen et al., 2016). Repiso et al. (Gutierrez-Repiso et al., 2020) analyzed gut microbiota composition in 46 patients with low Home-IR, high Home-IR, and T2DM patients treated with metformin. The results showed that compared with the low HOMA-IR group, the high HOMA-IR group had significantly higher flora abundance (q5.011) in Proteus (W52), Fusobacterium (W52), and Bacteroides (W51). It was also found that some gut microbiota, such as Prevotella copri and Bacteroides vulgatus, can affect the insulin resistance of diabetic patients by synthesizing branched-chain amino acids (BCAAs)-leucine, isoleucine, and valine (White and Newgard, 2019). In contrast, the content of BCAAs in the serum metabolic group of patients with insulin resistance increases, and the increase in BCAAs intake in food is associated with a higher risk of insulin resistance. Reducing BCAAs intake can improve postprandial insulin sensitivity, so BCAAs intake is considered an indicator of insulin resistance and a predictor of diabetes development (Shou et al., 2019). Thus, we can conclude that the change in gut microbiota can affect the insulin resistance of diabetic patients.

### Diabetic patients experience an inflammatory state caused by a disruption in their gut microbiota

Diabetic patients are in a chronic inflammatory state. T1DM is a proinflammatory problem, leading to islet βcell crushing and the loss of insulin production (Rodriguez-Valera et al., 2009). In T2DM, the proinflammatory state can lead to insulin resistance (Scheithauer et al., 2016), and the gut microbiota can mediate the occurrence and development of this inflammatory state in many ways. First of all, lipopolysaccharide (LPS) is one of the components of the outer membrane of gram-negative bacteria. LPS and LPS cytokines, such as IL-1 and IL-6, can combine with their toll-like receptor 4 (TLR4) to increase proinflammatory molecules. The receptor is found in cells from a variety of organs and tissues, including human adipose tissue, the brain, the liver, muscle, and the pancreas. Therefore, when the intestinal gram-negative bacteria change, it can affect the inflammatory state in the body. At the same time, some cytokines, such as IL-10 and IL-22, can play an anti-inflammatory role, while Enterobacter, Bacteroides fragilis, Achmania mucophilus, and Lactobacillus plantarum can induce the production of these cytokines (Zhu et al., 2018; Chen et al., 2022). Secondly, GM can affect intestinal barrier function. LPS can destroy the tight junction between epithelial cells, thus reducing the tight junction proteins (occludin and occlusive zone-1) and CB2 (Hasain et al., 2020). The decomposition products of GM can be used as the energy substrate of the intestinal epithelium to promote the renewal metabolism and damage repair of the intestinal epithelium (De Vadder et al., 2014). Short-chain fatty acids (SCFAs), mainly propionic acid, butyric acid, etc.), the products of cellulose and carbohydrate decomposed by GM, can help maintain the integrity of the intestinal epithelium by inducing mucin synthesis and improve the intestinal barrier by promoting tight connection assembly (Burger-van Paassen et al., 2009). When the balance of gut microbiota is broken, the intestinal barrier function is reduced, which can leak whole bacteria, fatty acids, and lipopolysaccharides and transfer them to all body parts through blood transport. Thus, TLR4 is activated, which leads to metabolic inflammation and accelerates the progression of diabetes (Que et al., 2021). Meanwhile, bacteria entering the body stimulate the immune system and produce antibodies against them. These antibodies will cross-react with islet cell surface antigens, and the cross-reaction of T cells will mediate the destruction of islet cells and the formation of T1DM (Cole et al., 2016). In addition, the anti-inflammatory properties of short-chain fatty acids can also be shown by directly inhibiting the transport of harmful bacteria through epithelial cells (Macfarlane and Macfarlane, 2011). Butyrate can regulate the function of macrophages to reduce the expression of proinflammatory mediators, promote the differentiation of regulatory T cells, and thus enhance anti-inflammatory properties (Qin et al., 2016). Besides, GM makes the host resistant to the colonization of pathogenic bacteria by occupying the host niche, which plays a vital role in preventing infection (Backhed et al., 2012).

### Gut microbiota affects energy intake and absorption

Gubat et al. sequenced and analyzed the GM of normal-weight children and overweight children respectively (Golloso-Gubat et al., 2020). The results showed that Bifidobacterium, Turicibacter, and Clostridiaceae were higher in normal-weight children, and Lachnospira was higher in overweight children. Studies have shown that inulin and other prebiotic fibers can prevent overeating related to energy-intensive diet intake in rodents (Chassaing et al., 2015). We can see that GM and its metabolites can affect the energy intake and absorption of T1DM patients through multiple channels, which can affect the appetite and total energy intake of patients as well as the consumption and metabolism of carbohydrates, fats, and other dietary components through peripheral and central channels (Rowland et al., 2018). In the gastrointestinal tract, GM and its metabolites, such as SCFAs, Peptide Y(PYY) and indole derivatives, can combine with vagal afferent neurons to transmit information to the nucleus tractus solitarius to affect the body’s sense of satiety (Tolhurst et al., 2012; Raybould and Zumpano, 2021). In addition, GM can also improve the sensitivity of the body to leptin by affecting the release of cholecystokinin (CCK) and glucagon-like peptide-1 (GLP-1), and then affect satiety through the gut-brain axis (Kałużna-Czaplińska et al., 2017). Some anti-diabetes drugs have been proven to be able to control patients’ blood glucose through the above ways (Holmes, 2016). Besides, we have mentioned that when the GM of patients is disordered, the body will be in an inflammatory state. This inflammatory state will also appear in the central solar tract and hypothalamus, thus affecting gut-brain feedback, appetite, and energy consumption, which may be related to microglia in the central system (Heiss and Olofsson, 2018). GM can also regulate the reward pathway of the central nervous system. Carbohydrate compounds can bring pleasure to people by promoting the production of dopamine, allowing people to increase their intake of carbohydrate foods. Inulin can reduce the activation of reward-related regions in the brain, thus reducing the attraction of carbohydrates to the body, thereby reducing their intake (Walker et al., 2018). In addition, GM can also regulate adipose tissue distribution and vitamin synthesis (Rinninella et al., 2019; Kumar et al., 2020).

### The interaction between gut microbiota and the immune system

The pathogenesis of both T1DM and T2DM involves the immune system, including inflammation and autoimmunity dysfunction (Moffa et al., 2019). The abnormality of the immune system plays a significant role in the occurrence and development of T1DM, and the gut microbiota plays a crucial role in the regulation and function of the immune system (Salazar et al., 2020). After the appearance of T1DM-related autoantibody, the GM produced insufficient butyric acid-producing bacteria, and the bacterial diversity and community stability were low (Dedrick et al., 2020). de Goffau et al. (2013) performed pyrophosphate sequencing of the fecal specimens retained from 18 children positive for at least two diabetes-related autoantibodies while setting up a control group of 18 healthy children to match them. Compared to the autoantibody-negative group, the two most predominant bifidobacterial species observed in the observation group, namely Bifidobacterium adolescentis and Bifidobacterium pseudostreptum were deficient. At the same time, the number of Bacteroidetes spp. was increased. Bell et al. (2022) concluded that remodeling the GM can significantly affect the immune system of patients with T1DM. Therefore, we can see that GM is related to the autoimmune status of diabetic patients, and the adaptive immune system of patients can be regulated by regulating GM. In addition, GM can affect the occurrence of diabetes by affecting the innate immune system. In the T1DM anode mouse model, the deletion of MyD88, the primary response gene for medullary differentiation of the innate immune adapter, provides disease-dependent protection for the microbiota: under sterile (GF) conditions, MyD88 negative mice, but under specific pathogen-free conditions, do not develop diseases; in GF mice containing multiple GM, colon cancer reduced the occurrence of T1DM in MyD88 negative but nonwild type NOD mice (Burrows et al., 2015). At the same time, GM can help the development of intestinal-associated lymphoid tissue and lymphocytes, which plays a vital role in lymphocyte function, leading to inflammation or immune tolerance (Kamada et al., 2013). Under normal circumstances, immune cells such as macrophages in the body have low reactivity to normal symbiotic bacteria in the intestinal tract and will not produce an obvious proinflammatory reaction. GM plays a vital role in this immune tolerance. When GM is dysfunctional, some bacteria can induce the expression of IL-1β, leading to the disorder of the immune system in the body (Franchi et al., 2012) and accelerating the progression of diabetes. In addition, GM can also make immune cells react faster to infection by regulating the immune response in the intestine to maintain homeostasis in the intestine (Gomes et al., 2018).

**Figure 1:**
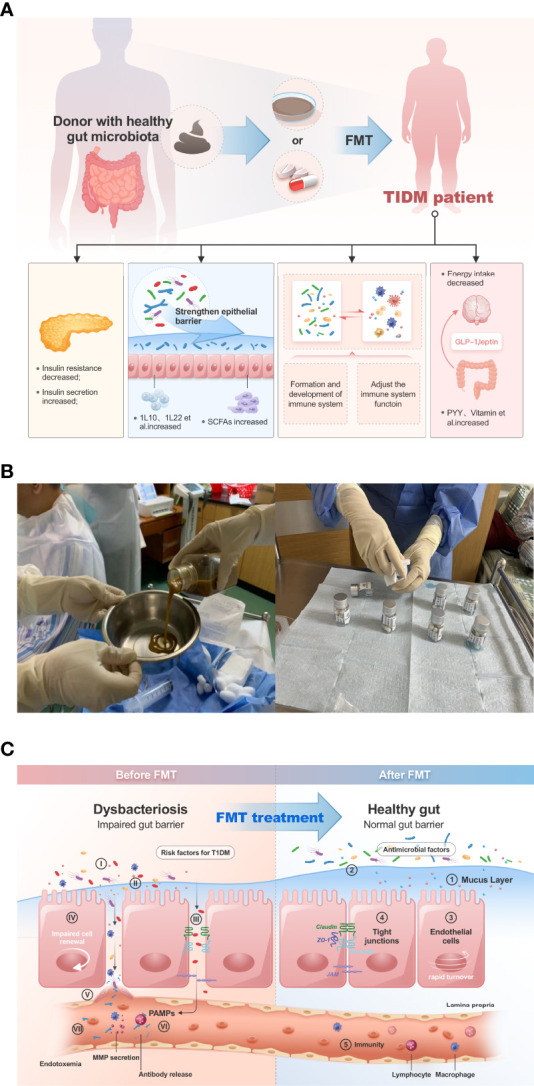
**(A)** FMT can improve the disease progression of T1DM patients in several ways. **(B)**, in our clinical trial study, patients were given gut microbiota transplantation by injection of bacterial solution and oral capsules (Clinical Registration Number: ChiCTR2100045789, Chinese Clinical Trail Registry: http://www.chictr.org.cn/showprojen.aspx?proj=125179). **(C)**, FMT improves dysbiosis of gut microbiota in type 1 diabetic patients.

Above all, we can see the critical role of GM in the development of diabetes, it is also the mechanism of fecal microbiota transplantation in the treatment of T1DM (**Figure 1A**). Fecal microbiota transplantation (FMT) have been conducted to treat various internal and external intestinal diseases by oral probiotics, prebiotics with satisfactory results (Al-Jameel, 2021).

## Feasibility of FMT in the treatment of T1DM patients

### FMT can help to modify blood glucose and insulin resistance

FMT is an aggressive and effective therapeutic approach to alter the microbiome in a limited clinical setting. FMT involves the transfer of gut microbiota from a healthy donor screened for pathogens to a recipient through oral capsules, enemas, or transnasal intestinal tube infusion of bacterial fluids, thus providing colonization resistance, producing beneficial metabolites, and restoring interaction with the mucosal immune system interactions (Sorbara and Pamer, 2022). During FMT, the role includes not only GM but also virulent fungi in the bacterial fluid, metabolites of the GM, the restoration of the mucosal immune system, and short-chain fatty acids play a crucial role (Leiva-Gea et al., 2018). Mankind has never stopped exploring FMT for the treatment of internal and external intestinal diseases from ancient times to the present. According to ancient biblical records, as early as 3000 years ago, some Indians applied cow dung to treat gastrointestinal diseases; in 400 BC, Chinese medical sage Li Shizhen used “Huang Long Tang” (a mixture of fresh feces and water) to treat patients with chronic diarrhea (Oprita et al., 2016). In Western countries, gut microbiota transplantation has also been explored for the treatment of intestinal and external diseases since the mid-20th century (Khoruts et al., 2015). In the past, due to limited medical technology, the treatment of FMT was initially performed by transplanting the whole feces of a healthy person into the patient, i.e., “swallowing feces by mouth”. It seemed to be an unacceptable treatment for many people. This treatment was also not in line with the concept of precision medicine, and there were infections and immune rejection for the transplanted patients. It is also a risk of infection and immune rejection for transplant recipients (Halaweish et al., 2022). Later, with the development of microbiology, people started to culture colonies that might benefit patients, but many bacteria and fungi require harsh culture environments and long culture cycles, which also hindered the development of colony transplantation techniques. It was not until the emergence of biotechnologies such as macrogenome sequencing, 16sRNA, and human gut microarrays that the study of gut microbiota became more operational and free from dependence on bacterial culture (Lee and Rho, 2022); the analysis of gut microbiota became more accurate and comprehensive, and the analysis of flora could be studied down to the species level and even the strain level of microorganisms, and the interactions between different species of microorganisms, the association between microorganisms and the environment, etc. could be explored. This has led to the rapid development of FMT technology (Org et al., 2017). In 2011, Time Magazine listed flora technology as one of the top ten breakthroughs in medicine, and since then more research has been conducted around FMT for the treatment of diseases.

The basic steps of FMT are as followings. First is the selection of donor and recipient. Recipients have different inclusion and exclusion criteria according to different research categories, but the general exclusion principles are severe cardiac, hepatic, renal, and other vital organ insufficiencies; leukopenia, the manifestation of autoimmune disease or diagnosed autoimmune disease within the past three months; other gastrointestinal diseases that may affect drug absorption; use of other hormones, antibiotics, and patients treated with probiotic prebiotics (He et al., 2022). Regarding the selection of flora donors, the inclusion criteria for autologous donors are equivalent to the inclusion criteria for recipients. As for allogeneic donors, it is necessary to exclude that they have hereditary diseases, autoimmune diseases, infectious diseases, diabetes, and gastrointestinal diseases, have not taken hormones, antibiotics and proton pump inhibitors in the past 3 months, and have not received vaccines and other tested drugs in the past 6 months (Zhang et al., 2019). Donors also should have a good and healthy psychological state, free from anxiety, depression and traumatic stress, and can be assessed by the international self-rating anxiety scale (SAS) and self-rating depression scale (SDS) (Mollayeva et al., 2016). Allogeneic donors are required to have a body mass index (BMI) less than 30 Kg/m2 (Woodworth et al., 2017). Allogeneic donors should be older than 18 years of age. There are no special rules regarding the upper age limit, but elderly people are excluded because of the combination of chronic diseases such as dysfunction of vital organs and diabetes. There is no special requirement for donor gender, but the donor should maintain good lifestyle habits, such as a reasonable diet (regular eating and healthy diet structure) and moderate exercise (Anand et al., 2017). Stool samples collected during pre-FMT matching and post-FMT clinical follow-up are often preserved in preservative solution, which is suitable for clinical studies because of the low requirements for equipment and the preservation environment. There is a special reagent tube for collecting stool samples from patients, which contains stool sample preservation solution and a sampling spoon. After sampling with the sampling spoon, put the fecal sample into the bottom of the sampling tube so that the fecal sample is completely immersed in the sample storage solution, and then screw the tube cover tightly and shake it well. The fecal sample can be stored at room temperature or in a household refrigerator for up to 12 months. Among them, there are many kinds of fecal preservation solutions, and the commonly used preservatives are ethanol, RNAlate, EDTA salt, sodium citrate, and other substances (Guan et al., 2021). The next step is the testing of blood samples and stool specimens from the donor, and these basic tests can be done in general hospital outpatient clinics. After the screening of the donor and recipient, the second step of FMT is to analyze the fecal specimens of both parties, type them, and make the flora capsules, liquid or oil. Stool specimens prepared for FMT were preserved in Maltodextrin-trehalose containing cryoprotectants and then stored in a standard freezer at -80°C. Preservation did not require strict anaerobic conditions, only the removal of air above the specimen. Stool specimens were analyzed by 16S rRNA, metabolomic fingerprinting, and flow cytometry assays to retain optimal recovery potential over a 3-month observation period (Burz et al., 2019).Then the flora preparations are resuscitated in a water bath to about 37°C before transplantation to avoid discomfort to the patient (Smits et al., 2018). The method of flora transplantation usually includes oral capsules, nasogastric tube, and nasojejunal tube injection of bacterial solution or oil, etc. After the transplantation, clinicians need to observe the patients for any adverse reactions and follow up with the patients for a certain period to observe the clinical benefits and gut microbiota changes after receiving FMT (**Figure 1B**).

FMT is now used to treat a variety of diseases. First, FMT is a safe treatment modality, and in all available clinical cases of FMT, the most common adverse effects are mild clinical symptoms, including diarrhea, gastrointestinal cramps, nausea, bloating, flatulence, constipation, and fever (Allegretti et al., 2019). Second, FMT can effectively alter the gut microbiota of patients, and the effects of flora alteration have been found to be sustained in later clinical follow-ups. It was found in FMT for recurrent C. difficile infection that the distribution of GM in transplanted recipients may be broadly similar to that of healthy donors, and this effect has been shown to persist for up to one-year (Weingarden et al., 2015). In a randomized double-blind trial, 22 obese patients were enrolled in the study and divided into two groups receiving capsule FMT and placebo capsules, and the results could be seen in the patients receiving the capsule FMT group sustained changes in the gut microbiome and bile acid profile that were similar to those of the lean donor (Allegretti et al., 2020). FMT has shown significant clinical efficacy in the treatment of a variety of diseases, and in the treatment of intestinal diseases, FMT has an internationally recognized role in the treatment of refractory C. difficile infections, with efficiency rates exceeding those of advanced antibiotics such as vancomycin and cure rates of more than 85%, FMT becomes a recommended therapy for recurrent CDI by the American College of Gastroenterology and the Infectious Diseases Society of America (Smillie et al., 2018). Given the close relationship between GM and the endocrine and immune systems, more and more studies have focused on immuno-metabolic and diabetes-related diseases. It has been found that the structure and function of the intestinal barrier can be restored by FMT, which can alleviate the chronic inflammatory state in diabetic patients, improving their clinical symptoms and slowing down the progression of the disease (Ganesan et al., 2018).

Many studies have found that FMT significantly improves insulin resistance, islet secretion, and dysbiosis in mice with non-obese diabetes (NOD) (Vrieze et al., 2012). We have already mentioned that FMT can restore short-chain fatty acids in the intestine (Allegretti et al., 2019), which we have already mentioned can control the progression of diabetes in several ways (Hanssen et al., 2021). FMT can also improve insulin sensitivity and control the progression of diabetes by affecting the autoimmune status of patients (Allegretti et al., 2020). The bacteriophage component of the GM can enter the brain through the intestinal and blood-brain barriers (Chen et al., 2022). Gabanyi et al. (2022) studied mice lacking the pattern recognition receptor Nod2, it was found that intestinal bacterial cell wall debris can cross the intestinal barrier into the brain through the blood circulation and bind to Nod2 in specific neurons in the hypothalamus thereby regulating appetite and body weight; in addition, GM can affect the autonomic nerves in the gut leading to changes in satiety and mood, thus FMT can modulate the patients’ gut-brain axis to control insulin resistance and body weight (Hartstra et al., 2020). It was also found that the SCFA-producing microbiota was reduced in T1DM mice (Hanssen et al., 2021), and the addition of propionic acid-producing mucilaginous Ackermania or probiotics that significantly increased SCFA production to the gut of non-obese diabetic (NOD) mice resulted in a reduced incidence of T1DM in NOD mice; Hui W et al. (Wang et al., 2019) performed FMT on a T2DM mouse model established by a high-fat diet combined with streptozotocin and found that insulin resistance and islet β-cell function were improved after FMT, and the inflammatory response of mouse pancreatic tissue was also decreased and apoptosis of islet β-cells was somewhat inhibited. Enterobacteriaceae is a genus of opportunistic endotoxin-producing pathogenic bacteria in mice, with 35% of the gut bacteria in morbidly obese volunteers with diabetes and severe metabolic disorders (Cani et al., 2007). Fei N et al. (Fei and Zhao, 2013) organized a 23-week cereal plus probiotic diet for volunteers. The result showed that after the intervention, the volunteers’ body weight was effectively reduced; the abundance of Enterobacteriaceae was reduced from 35% to undetectable, and hyperinsulinemia, insulin resistance, and hyperglycemic states were alleviated. To some extent, probiotic intake also belongs to FMT, and this study mentions the effectiveness of FMT in improving glycemic control and insulin resistance in patients. A case has been reported in which a female patient with an 8-year history of diabetes mellitus with poor glycemic control under the control of glucose-lowering medication had an excellent clinical response and control of glycemia and related diabetic complications after receiving two FMTs within 3 months (Cai et al., 2018). In the treatment of a study enrolling 38 patients with metabolic syndrome, who were divided into two groups receiving allogeneic FMT from lean donors and autologous FMT from their fecal infusion, it was found after 6 weeks that patients receiving allogeneic FMT had increased insulin sensitivity and that this clinical change may be associated with an increase in mucinous Ackermania in the intestine (Kootte et al., 2017). Mocanu et al. found in a randomized, double-blind controlled trial of 61 patients with obesity and metabolic syndrome, FMT supplemented with low fermentable fiber significantly improved insulin resistance in patients and that this metabolic benefit was associated with improved intestinal endocrine function, altered GM abundance, and increased donor bacteria (Mocanu et al., 2021). Above all, FMT can improve the disease progression of diabetic patients in several ways (**Figure 1C**).

### Our study

Two groups of subjects underwent autologous FMT and allogeneic FMT in a randomized controlled trial of new-onset T1DM patients within 6 months; the results showed that FMT stabilized residual -β cell function and optimized glycemic control in patients with new-onset T1DM and that the GM of patients changed at the phylum, genus, and species levels, with a lot of flora such as D. pigerand, B. stercoris, Prevotella spp, and S. oralis correlating with the progression of the treatment course correlated (de Groot et al., 2021). Based on the known metabolic benefits of FMT in patients with autoimmune type 1 diabetes, we treated two adolescent patients with autoimmune type 1 diabetes with FMT (He et al., 2022). First, we performed multiple FMT at different nodes in two T1DM patients; second, we followed both patients clinically for 34 and 19 weeks, respectively, during which stool and serum samples were collected. No adverse events were observed in either patient during our clinical study. Based on the macrogenome sequencing of the stool samples, we concluded that FMT resulted in the colonization of beneficial bacteria in T1DM patients and that the colonization of these flora persisted during the long-term follow-up after the finish of the FMT treatment. Based on the colonization of beneficial bacteria, the clinical outcomes of both patients were significantly improved, and they stopped the use of insulin and some oral hypoglycemic agents. Their blood glucose levels remained. The clinical outcomes were also significantly improved in two patients who had discontinued insulin and some oral hypoglycemic agents, and whose blood glucose levels remained at a more optimal level. Although the number of patients included in this clinical trial is small, it provides strong theoretical and practical support for us to conduct more clinical research and treatment of T1DM patients with FMT. In addition, we also identified several characteristic bacteria that may be related to the progress of T1DM. According to the analysis of the correlation between the gut microbiota and clinical indicators of patients at the level of genus and species, Faecalibacterium and Butyricimonas were negatively correlated with the insulin resistance (Homa IR) of patients, while Blautia and Anaerostipes were positively correlated with insulin resistance. P. Successives, P. faecium may improve the insulin secretion of patients. L. bacterium GAM79, Clostridium bone, and B. caccae are negatively correlated with insulin secretion index.

## Current development status of FMT

### Safety and Limitations of FMT

Several studies have shown that FMT appears safe, and patients are less likely to experience adverse reactions. A meta-analysis that included 61 studies after searching to identify 378 reference articles (Rapoport et al., 2022) showed that less than 1% of the 5099 patients who underwent FMT experienced FMT-related serious adverse effects (SAEs). However, on June 13, 2019, the US Food and Drug Administration (FDA) issued a warning about the risks of FMT when they reported two cases of patients who transferred antibiotic-resistant microorganisms [specifically, broad-spectrum β-lactamase-producing Escherichia coli (E. coli)] *via* FMT, causing the patients to develop transplant-related sepsis and leading to death in one of the patients (Battaglioli et al., 2018), in which none of the donor’s stool was screened for this resistant antibiotic, and the recipients were immunocompromised patients. DeFilippo et al. (DeFilipp et al., 2019) also reported drug-resistant E. coli bacteremia transmitted by fecal microbiota transplantation.

With the introduction of macroeconomic analysis, it has become clear that, in addition to bacteria, the fecal microbiota contains considerable numbers of viruses, fungi, and phages, as well as intact shed colonic cells. One study reported that feces contained 1011 bacteria/g, 107 intact colonocytes/g, 108 viruses/g, and 108 archaea/g. Although bacteria dominate the intestinal population, other components, such as viruses and miRNAs, cannot be excluded from influencing host physiology (Liu et al., 2016). Since the transfer of unidentified microbial communities may pose some risk, Ott et al. asked whether sterile fecal microbial filtrates would also have beneficial biological effects and investigated this. Their team performed FMT on five patients with relapsed CDI using a filtered (small particles and bacteria removed) fecal solution and found that this sterile (containing bacterial debris, proteins, DNA, antimicrobial compounds, metabolites, and viruses) fecal microbial filtrate could alter the patients’ gastrointestinal microbiota and eliminate their gastrointestinal symptoms (Ott et al., 2017), suggesting that non-bacterial elements may play a more important role than previously recognized. In line with this, Zuo et al. (2018) recently reported that phage transfer during FMT could affect the progression of CDI. Similarly, Conceiço-Neto et al. (Conceicao-Neto et al., 2018) suggested that eukaryotic viral groups are associated with successfully treating ulcerative colitis by FMT. These studies suggest that bacterial fractions, metabolites, or phages can mediate the transfer of whole fecal microorganisms. Therefore, in gut microbiota transplantation, non-bacterial components of donor feces may also be transferred through FMT, and the role that these known and unknown components play in transplantation and the impact they bring are also unknown.

Likewise, the development of FMT in the clinical setting is often limited. Firstly, some patients and even clinicians have questioned the effectiveness of FMT in treating T1DM, which greatly hinders the development of therapeutic studies of FMT in the clinical setting. Secondly, many autoimmune-mediated T1DM patients are adolescents, and it is not ethical to conduct clinical studies on patients who are too young. For older minor patients, their lack of cooperation with FMT treatment, lifestyle modifications such as diet structure, late clinical follow-up, and the lack of awareness of their patients also make it more difficult to develop the technology. In addition, we have already mentioned that GM can be affected by many factors, such as the external environment (different air quality and composition of drinking water), different dietary structures, the use of probiotics, antibiotics, and personal habits of smoking and drinking (Hanssen et al., 2021). This leads to a certain degree of limitation of the clinical data and conclusions from the existing FMT clinical studies for the reference of subsequent studies. The clinical benefits of GM for one region or even for one patient may not necessarily be the same for other patients.

### Lack of consistency in the supervision of FMT

The European Commission decided that member countries are free to regulate FMT at the national level, which has led to regulatory haphazardness among member states and even a lack of any regulatory standards for FMT in some countries (Verbeke et al., 2017). Lack of standardized regulation can create confusion, while overly restrictive regulation may hinder access to fecal bacteria and research on FMT (Allegretti et al., 2019). Restricting the use of FMT through regulation may lead to some unintended consequences. It becomes less difficult to perform FMT in an environment without medical supervision. People can perform FMT with impunity, and patients can search the Internet for instructions and methods of home FMT and perform self-transplantation, leading to a significant increase in the number of self-FMT (Segal et al., 2018). The lack of regulation, its unclear donor source, the acquisition process, and the lack of rigor in the transplantation process can significantly increase the risk of transplantation-related diseases (e.g., infectious diseases). On the other hand, the excessive regulatory restrictions have also caused significant problems for those wishing to conduct clinical studies on FMT, and the complex applications and approvals required to conduct clinical trials have discouraged many researchers, incredibly discouraging many clinical workers and preventing them from engaging in such studies (Bunnik et al., 2017).

### The effectiveness and efficiency of FMT cannot be determined

Microbial diversity was found to be a reliable predictor of FMT success by comparing the gut flora characteristics of different donors (Kump et al., 2018). Patients who achieve a clinical response to FMT generally show higher microbial diversity than non-responders (Paramsothy et al., 2017b). We suggest that the success of FMT can be considered a two-step process: first, requiring transplanting the transplanted microbiome into a new host and increasing the local commensal community, and then clinical improvement may be observed. The selection of a suitable fecal donor is a critical factor in the success of FMT (Vermeire et al., 2016). However, other factors, such as genetics and environment can also influence the success of FMT. It has been suggested that remission rates can be improved by pooling donor stools together, thereby limiting patients’ chances of receiving only ineffective stools (Kazerouni and Wein, 2017). This approach was studied in a cohort of 85 patients with mild to moderate ulcerative colitis in Australia (Paramsothy et al., 2017a), and patients in the treatment group received a mixture of stools containing up to seven different donors in the hope that the donor-dependent effects could be homogenized. In addition, a more intensive dosing regimen was used, with initial FMT by colonoscopy followed by fecal enemas five times a week for eight weeks. Despite the multiple donors and intensive dosing approach, Paramsothy et al. achieved a remission rate after FMT (27% for FMT versus 8% for placebo, p = 0.02), similar to that reported previously. Therefore, although the effectiveness of FMT is related to microbial diversity, simply increasing the biodiversity of the donor does not guarantee the effectiveness of transplantation.

## Discussion

Autoimmune-mediated T1DM patients have a significant alteration of gut microbiota, and gut microbiota, as the “second genome” of humans, can affect the disease progression of T1DM in many ways (Abdellatif and Sarvetnick, 2019). FMT can improve the glycemic control and insulin resistance of T1DM patients by adjusting the imbalance of gut microbiotaT1DM patients. Many studies have shown that GM is closely related to the occurrence and development of T1DM. In a case-control study, the feces of 16 T1DM children and 16 healthy children were analyzed for GM. It was found that there were significant differences in gut microbial structure between the two groups of children. The abundance of actinomycetes and chlamydia, and the proportion of chlamydia and Bacteroides in T1DM children were lower than those in healthy children, and the number of Clostridium, Bacteroides, and micro-venous bacilli in the intestine of T1DM children was higher (Murri et al., 2013). It was also found that the microbiome of healthy children was more diversified and stable than that of children with T1DM. After the occurrence of autoimmune diseases, the level of Firmicum decreased, and the level of bacteroids increased (Giongo et al., 2011). Stewart et al. (2018) analyzed the fecal specimens through 16s rRNA and macroeconomic sequencing retained from 903 patients with T1DM aged 3 months to 46 months and the healthy control group patients; the abundance of genera of bacteria changed between the observation and control groups. They found higher levels of streptococcus and lactococcus in T1DM. It could protect the intestinal mucosal barrier function, enhance intestinal integrity, and reduce the chronic inflammatory state in diabetic patients (Brown et al., 2011). Many drugs have induced changes in the GM of diabetic patients while controlling blood glucose. Metformin is the first-line cornerstone drug for glycemic control in diabetic patients, and Bryrup et al. (2019) found that after six weeks of continuous oral administration of metformin, patients experienced significant changes in gut microbiota richness, with a decrease in the abundance of Enterobacter spp. and Clostridium spp. and an increase in the abundance of Salmonella spp. and Shigella spp. and Biliophage spp. Gu et al. (2017) demonstrated that acarbose treatment altered the composition of the GM, with increased concentrations of Lactobacillus and Bifidobacterium and decreased concentrations of Bacteroidetes and Clostridium, both of which could improve insulin resistance in patients by modulating bile acid metabolism. Therefore, we can see that T1DM patients do have gut microbiota disorders and the improvement of blood glucose levels in diabetic patients is closely related to the change in gut microbiota. The effectiveness of FMT in improving patients’ glycemic control and insulin resistance can also be seen in the clinical treatments around FMT listed in the previous section, but there are still two major problems. Firstly, many clinicians are skeptical about FMT for T1DM, they believe that gut microbiota plays a limited role in the progression of T1DM and deny that FMT can bring significant glycemic control benefits to T1DM patients, and they believe that the safety of FMT is still to be investigated, which largely hinders the development and application of FMT treatment technology. Secondly, the clinical studies on the treatment of T1DM by FMT are limited, and there is a lack of large-scale clinical trials with many subjects and no case-control trials with different nodes transplanted for comparison, and the mode, number, and time point of transplantation have not yet been fully standardized. We have not yet demonstrated how often and how many repeat transplants are needed to achieve the best clinical benefit for patients. We also cannot specifically control the effect of other food medications on the efficacy of FMT. Many questions need to be addressed in the future development of FMT treatment technology.

In the future development of FMT treatment technology, many questions must be solved. Firstly, its safety should be further improved. The technology should be able to effectively screen out pathogenic intestinal bacteria and try to avoid serious adverse events, such as infection, aggravation of gut microbiota dysbiosis, and immune rejection-related events. Secondly, the National Health Organization should establish and improve the standardized system of FMT for disease treatment and clarify the relevant indications, contraindications, transplantation methods, and related expenses. Third, the implementation of “precise transplantation technology” is the transplantation of specific disordered microbiota related to the disease to restore normal intestinal homeostasis and then regulate the immune and metabolic disorders in the patient’s body. Fourthly, we should be aware of the importance of clinicians in the development of FMT for T1DM. Therefore, FMT-related studies could be added to the mini-lessons in hospitals to complement the knowledge gaps of clinicians in this field, so that they can see the clinical benefits of FMT for T1DM patients and thus facilitate more therapeutic studies. Fifth, at present, the specific mechanism of FMT in the treatment of autoimmune-mediated T1DM has not been completely revealed. Many researchers believe that the benefits of FMT in T1DM patients such as the change of autoimmune status and the improvement of beta-cell function are related to SCFAs (Jacob et al., 2020). However, another study found that oral SCFAs did not improve the innate immunity and islet autoimmunity of T1DM patients (de Groot et al., 2020). Therefore, the mechanism of FMT intervention in the treatment of T1DM remains to be further explored. Finally, there are still many questions about the transplantation process: for example, how can oral capsules or nasal-intestinal tube injections achieve better therapeutic effects through the transplantation method? Are multiple transplants better than single transplants? If multiple transplants are performed, what is the optimal time interval between each transplant, the duration of FMT, and how to extend the duration of treatment? How much will the use of antibiotics, hormones, immunosuppressants, and other probiotics affect FMT’s efficacy, and can the adverse effects be avoided? These unknowns will hopefully be further addressed in the future development of FMT technology.

## Author contributions

Conceptualization, KH and LL. Writing—original draft preparation, SZ, ZW, and FC. Writing— review and editing, FD and JC. Supervision, KH. Funding acquisition, LL. All authors have read and agreed to the published version of the manuscript.

## Funding

This work was supported by grants from the Guangdong Science and Technology Special Fund (No.210629086900260), Longhu People’s Hospital, Shantou, China.

## Conflict of interest

The authors declare that the research was conducted in the absence of any commercial or financial relationships that could be construed as a potential conflict of interest.

## Publisher’s note

All claims expressed in this article are solely those of the authors and do not necessarily represent those of their affiliated organizations, or those of the publisher, the editors and the reviewers. Any product that may be evaluated in this article, or claim that may be made by its manufacturer, is not guaranteed or endorsed by the publisher.

## References

[B1] Abdellatif A. M. Sarvetnick N. E. (2019). Current understanding of the role of gut dysbiosis in type 1 diabetes. J. Diabetes 11 (8), 632–644. doi: 10.1111/1753-0407.12915 30864231

[B2] Al-Jameel S. S. (2021). Association of diabetes and microbiota: An update. Saudi J. Biol. Sci. 28 (8), 4446–4454. doi: 10.1016/j.sjbs.2021.04.041 34354429 PMC8324937

[B3] Allegretti J. R. Kassam Z. Mullish B. H. Chiang A. Carrellas M. Hurtado J. . (2020). Effects of fecal microbiota transplantation with oral capsules in obese patients. Clin. Gastroenterol. Hepatol. 18 (4), 855–63.e2. doi: 10.1016/j.cgh.2019.07.006 31301451

[B4] Allegretti J. R. Mullish B. H. Kelly C. Fischer M. (2019). The evolution of the use of faecal microbiota transplantation and emerging therapeutic indications. Lancet 394 (10196), 420–431. doi: 10.1016/S0140-6736(19)31266-8 31379333

[B5] American Diabetes Association Professional Practice C. 2 (2022). Classification and diagnosis of diabetes: Standards of medical care in diabetes-2022. Diabetes Care 45 (Suppl 1), S17–S38. doi: 10.2337/dc22-S002 34964875

[B6] Anand R. Song Y. Garg S. Girotra M. Sinha A. Sivaraman A. . (2017). Effect of aging on the composition of fecal microbiota in donors for FMT and its impact on clinical outcomes. Dig Dis. Sci. 62 (4), 1002–1008. doi: 10.1007/s10620-017-4449-6 28181098

[B7] Backhed F. Fraser C. M. Ringel Y. Sanders M. E. Sartor R. B. Sherman P. M. . (2012). Defining a healthy human gut microbiome: current concepts, future directions, and clinical applications. Cell Host Microbe 12 (5), 611–622. doi: 10.1016/j.chom.2012.10.012 23159051

[B8] Battaglioli E. J. Hale V. L. Chen J. Jeraldo P. Ruiz-Mojica C. Schmidt B. A. . (2018). Clostridioides difficile uses amino acids associated with gut microbial dysbiosis in a subset of patients with diarrhea. Sci. Transl. Med. 10 (464), eaam7019. doi: 10.1126/scitranslmed.aam7019 30355801 PMC6537101

[B9] Bell K. J. Saad S. Tillett B. J. McGuire H. M. Bordbar S. Yap Y. A. . (2022). Metabolite-based dietary supplementation in human type 1 diabetes is associated with microbiota and immune modulation. Microbiome. 10 (1), 9. doi: 10.1186/s40168-021-01193-9 35045871 PMC8772108

[B10] Brown C. T. Davis-Richardson A. G. Giongo A. Gano K. A. Crabb D. B. Mukherjee N. . (2011). Gut microbiome metagenomics analysis suggests a functional model for the development of autoimmunity for type 1 diabetes. PloS One 6 (10), e25792. doi: 10.1371/journal.pone.0025792 22043294 PMC3197175

[B11] Bryrup T. Thomsen C. W. Kern T. Allin K. H. Brandslund I. Jorgensen N. R. . (2019). Metformin-induced changes of the gut microbiota in healthy young men: results of a non-blinded, one-armed intervention study. Diabetologia. 62 (6), 1024–1035. doi: 10.1007/s00125-019-4848-7 30904939 PMC6509092

[B12] Bunnik E. M. Aarts N. Chen L. A. (2017). Physicians must discuss potential long-term risks of fecal microbiota transplantation to ensure informed consent. Am. J. Bioeth 17 (5), 61–63. doi: 10.1080/15265161.2017.1299816 28430073

[B13] Burger-van Paassen N. Vincent A. Puiman P. J. van der Sluis M. Bouma J. Boehm G. . (2009). The regulation of intestinal mucin MUC2 expression by short-chain fatty acids: implications for epithelial protection. Biochem. J. 420 (2), 211–219. doi: 10.1042/BJ20082222 19228118

[B14] Burrows M. P. Volchkov P. Kobayashi K. S. Chervonsky A. V. (2015). Microbiota regulates type 1 diabetes through toll-like receptors. Proc. Natl. Acad. Sci. U S A. 112 (32), 9973–9977. doi: 10.1073/pnas.1508740112 26216961 PMC4538618

[B15] Burz S. D. Abraham A. L. Fonseca F. David O. Chapron A. Beguet-Crespel F. . (2019). A guide for ex vivo handling and storage of stool samples intended for fecal microbiota transplantation. Sci. Rep. 9 (1), 8897. doi: 10.1038/s41598-019-45173-4 31222022 PMC6586871

[B16] Cai T. T. Ye X. L. Yong H. J. Song B. Zheng X. L. Cui B. T. . (2018). Fecal microbiota transplantation relieve painful diabetic neuropathy: A case report. Med. (Baltimore) 97 (50), e13543. doi: 10.1097/MD.0000000000013543 PMC6320208 30558014

[B17] Cani P. D. Amar J. Iglesias M. A. Poggi M. Knauf C. Bastelica D. . (2007). Metabolic endotoxemia initiates obesity and insulin resistance. Diabetes. 56 (7), 1761–1772. doi: 10.2337/db06-1491 17456850

[B18] Chafe R. Aslanov R. Sarkar A. Gregory P. Comeau A. Newhook L. A. (2018). Association of type 1 diabetes and concentrations of drinking water components in Newfoundland and Labrador, Canada. BMJ Open Diabetes Res. Care 6 (1), e000466. doi: 10.1136/bmjdrc-2017-000466 PMC5841498 29527309

[B19] Chassaing B. Miles-Brown J. Pellizzon M. Ulman E. Ricci M. Zhang L. . (2015). Lack of soluble fiber drives diet-induced adiposity in mice. Am. J. Physiol. Gastrointest Liver Physiol. 309 (7), G528–G541. doi: 10.1152/ajpgi.00172.2015 26185332 PMC4593822

[B20] Chen F. He L. Li J. Yang S. Zhang B. Zhu D. . (2022). Polyethylene glycol loxenatide injection (GLP-1) protects vascular endothelial cell function in middle-aged and elderly patients with type 2 diabetes by regulating gut microbiota. Front. Mol. Biosci. 9, 879294. doi: 10.3389/fmolb.2022.879294 35782875 PMC9240776

[B21] Chen F. Hou K. Chen Z. S. (2022). Gut microbes regulate the feeding center: a new discovery of gut brain axis. Signal Transduct Target Ther. 7 (1), 284. doi: 10.1038/s41392-022-01117-5 35963865 PMC9376083

[B22] Cole D. K. Bulek A. M. Dolton G. Schauenberg A. J. Szomolay B. Rittase W. . (2016). Hotspot autoimmune T cell receptor binding underlies pathogen and insulin peptide cross-reactivity. J. Clin. Invest. 126 (9), 3626. doi: 10.1172/JCI89919 PMC5004936 27525441

[B23] Conceicao-Neto N. Deboutte W. Dierckx T. Machiels K. Wang J. Yinda K. C. . (2018). Low eukaryotic viral richness is associated with faecal microbiota transplantation success in patients with UC. Gut. 67 (8), 1558–1559. doi: 10.1136/gutjnl-2017-315281 29066574 PMC6204959

[B24] Davis-Richardson A. G. Triplett E. W. (2015). A model for the role of gut bacteria in the development of autoimmunity for type 1 diabetes. Diabetologia. 58 (7), 1386–1393. doi: 10.1007/s00125-015-3614-8 25957231 PMC4473028

[B25] Dedrick S. Sundaresh B. Huang Q. Brady C. Yoo T. Cronin C. . (2020). The role of gut microbiota and environmental factors in type 1 diabetes pathogenesis. Front. Endocrinol. (Lausanne) 11, 78. doi: 10.3389/fendo.2020.00078 32174888 PMC7057241

[B26] DeFilipp Z. Bloom P. P. Torres Soto M. Mansour M. K. Sater M. R. A. Huntley M. H. . (2019). Drug-resistant e. coli bacteremia transmitted by fecal microbiota transplant. N Engl. J. Med. 381 (21), 2043–2050. doi: 10.1056/NEJMoa1910437 31665575

[B27] de Goffau M. C. Fuentes S. van den Bogert B. Honkanen H. de Vos W. M. Welling G. W. . (2014). Aberrant gut microbiota composition at the onset of type 1 diabetes in young children. Diabetologia. 57 (8), 1569–1577. doi: 10.1007/s00125-014-3274-0 24930037

[B28] de Goffau M. C. Luopajarvi K. Knip M. Ilonen J. Ruohtula T. Harkonen T. . (2013). Fecal microbiota composition differs between children with beta-cell autoimmunity and those without. Diabetes. 62 (4), 1238–1244. doi: 10.2337/db12-0526 23274889 PMC3609581

[B29] de Groot P. F. Belzer C. Aydin O. Levin E. Levels J. H. Aalvink S. . (2017). Distinct fecal and oral microbiota composition in human type 1 diabetes, an observational study. PloS One 12 (12), e0188475. doi: 10.1371/journal.pone.0188475 29211757 PMC5718513

[B30] de Groot P. F. Nikolic T. Imangaliyev S. Bekkering S. Duinkerken G. Keij F. M. . (2020). Oral butyrate does not affect innate immunity and islet autoimmunity in individuals with longstanding type 1 diabetes: a randomised controlled trial. Diabetologia. 63 (3), 597–610. doi: 10.1007/s00125-019-05073-8 31915895

[B31] de Groot P. Nikolic T. Pellegrini S. Sordi V. Imangaliyev S. Rampanelli E. . (2021). Faecal microbiota transplantation halts progression of human new-onset type 1 diabetes in a randomised controlled trial. Gut. 70 (1), 92–105. doi: 10.1136/gutjnl-2020-322630 33106354 PMC7788262

[B32] De Vadder F. Kovatcheva-Datchary P. Goncalves D. Vinera J. Zitoun C. Duchampt A. . (2014). Microbiota-generated metabolites promote metabolic benefits *via* gut-brain neural circuits. Cell 156 (1-2), 84–96. doi: 10.1016/j.cell.2013.12.016 24412651

[B33] Fei N. Zhao L. (2013). An opportunistic pathogen isolated from the gut of an obese human causes obesity in germfree mice. ISME J. 7 (4), 880–884. doi: 10.1038/ismej.2012.153 23235292 PMC3603399

[B34] Franchi L. Kamada N. Nakamura Y. Burberry A. Kuffa P. Suzuki S. . (2012). NLRC4-driven production of IL-1beta discriminates between pathogenic and commensal bacteria and promotes host intestinal defense. Nat. Immunol. 13 (5), 449–456. doi: 10.1038/ni.2263 22484733 PMC3361590

[B35] Gabanyi I. Lepousez G. Wheeler R. Vieites-Prado A. Nissant A. Wagner S. . (2022). Bacterial sensing via neuronal Nod2 regulates appetite and body temperature. Science 376 (6590), eabj3986. doi: 10.1126/science.abj3986 35420957

[B36] Ganesan K. Chung S. K. Vanamala J. Xu B. (2018). Causal relationship between diet-induced gut microbiota changes and diabetes: A novel strategy to transplant faecalibacterium prausnitzii in preventing diabetes. Int. J. Mol. Sci. 19 (12), 3720. doi: 10.3390/ijms19123720 30467295 PMC6320976

[B37] Giongo A. Gano K. A. Crabb D. B. Mukherjee N. Novelo L. L. Casella G. . (2011). Toward defining the autoimmune microbiome for type 1 diabetes. ISME J. 5 (1), 82–91. doi: 10.1038/ismej.2010.92 20613793 PMC3105672

[B38] Golloso-Gubat M. J. Ducarmon Q. R. Tan R. C. A. Zwittink R. D. Kuijper E. J. Nacis J. S. . (2020). Gut microbiota and dietary intake of normal-weight and overweight Filipino children. Microorganisms 8 (7), 1015. doi: 10.3390/microorganisms8071015 32650516 PMC7409305

[B39] Gomes A. C. Hoffmann C. Mota J. F. (2018). The human gut microbiota: Metabolism and perspective in obesity. Gut Microbes 9 (4), 308–325. doi: 10.1080/19490976.2018.1465157 29667480 PMC6219651

[B40] Guan H. Pu Y. Liu C. Lou T. Tan S. Kong M. . (2021). Comparison of fecal collection methods on variation in gut metagenomics and untargeted metabolomics. mSphere. 6 (5), e0063621. doi: 10.1128/mSphere.00636-21 34523982 PMC8550109

[B41] Gutierrez-Repiso C. Moreno-Indias I. Martin-Nunez G. M. Ho-Plagaro A. Rodriguez-Canete A. Gonzalo M. . (2020). Mucosa-associated microbiota in the jejunum of patients with morbid obesity: alterations in states of insulin resistance and metformin treatment. Surg. Obes. Relat. Dis. 16 (10), 1575–1585. doi: 10.1016/j.soard.2020.04.008 32475753

[B42] Gu Y. Wang X. Li J. Zhang Y. Zhong H. Liu R. . (2017). Analyses of gut microbiota and plasma bile acids enable stratification of patients for antidiabetic treatment. Nat. Commun. 8 (1), 1785. doi: 10.1038/s41467-017-01682-2 29176714 PMC5702614

[B43] Halaweish H. F. Boatman S. Staley C. (2022). Encapsulated fecal microbiota transplantation: Development, efficacy, and clinical application. Front. Cell Infect. Microbiol. 12, 826114. doi: 10.3389/fcimb.2022.826114 35372103 PMC8968856

[B44] Hanssen N. M. J. de Vos W. M. Nieuwdorp M. (2021). Fecal microbiota transplantation in human metabolic diseases: From a murky past to a bright future? Cell Metab. 33 (6), 1098–1110. doi: 10.1016/j.cmet.2021.05.005 34077717

[B45] Hartstra A. V. Schuppel V. Imangaliyev S. Schrantee A. Prodan A. Collard D. . (2020). Infusion of donor feces affects the gut-brain axis in humans with metabolic syndrome. Mol. Metab. 42, 101076. doi: 10.1016/j.molmet.2020.101076 32916306 PMC7536740

[B46] Hasain Z. Mokhtar N. M. Kamaruddin N. A. Mohamed Ismail N. A. Razalli N. H. Gnanou J. V. . (2020). Gut microbiota and gestational diabetes mellitus: A review of host-gut microbiota interactions and their therapeutic potential. Front. Cell Infect. Microbiol. 10, 188. doi: 10.3389/fcimb.2020.00188 32500037 PMC7243459

[B47] He L. Chen R. Zhang B. Zhang S. Khan B. A. Zhu D. . (2022). Fecal microbiota transplantation treatment of autoimmune-mediated type 1 diabetes mellitus. Front. Immunol. 13, 930872. doi: 10.3389/fimmu.2022.930872 36032108 PMC9414079

[B48] Heiss C. N. Olofsson L. E. (2018). Gut microbiota-dependent modulation of energy metabolism. J. Innate Immun. 10 (3), 163–171. doi: 10.1159/000481519 29131106 PMC6757175

[B49] Holmes D. (2016). Gut microbiota: Antidiabetic drug treatment confounds gut dysbiosis associated with type 2 diabetes mellitus. Nat. Rev. Endocrinol. 12 (2), 61. doi: 10.1038/nrendo.2015.222 26668122

[B50] Hou K. Wu Z. X. Chen X. Y. Wang J. Q. Zhang D. Xiao C. . (2022). Microbiota in health and diseases. Signal Transduct Target Ther. 7 (1), 135. doi: 10.1038/s41392-022-00974-4 35461318 PMC9034083

[B51] Hou K. Zhang S. Wu Z. Zhu D. Chen F. Lei Z. N. . (2021). Reconstruction of intestinal microecology of type 2 diabetes by fecal microbiota transplantation: Why and how. Bosn J. Basic Med. Sci. 22 (3), 315–325. doi: 10.17305/bjbms.2021.6323 PMC9162745 34761734

[B52] Jacob N. Jaiswal S. Maheshwari D. Nallabelli N. Khatri N. Bhatia A. . (2020). Butyrate induced tregs are capable of migration from the GALT to the pancreas to restore immunological tolerance during type-1 diabetes. Sci. Rep. 10 (1), 19120. doi: 10.1038/s41598-020-76109-y 33154424 PMC7644709

[B53] Kałużna-Czaplińska J. Gątarek P. Chartrand M. S. Dadar M. Bjørklund G. (2017). Is there a relationship between intestinal microbiota, dietary compounds, and obesity? Trends Food Sci. Technol. 70, 105–113. doi: 10.1016/j.tifs.2017.10.010

[B54] Kamada N. Seo S. U. Chen G. Y. Nunez G. (2013). Role of the gut microbiota in immunity and inflammatory disease. Nat. Rev. Immunol. 13 (5), 321–335. doi: 10.1038/nri3430 23618829

[B55] Kazerouni A. Wein L. M. (2017). Exploring the efficacy of pooled stools in fecal microbiota transplantation for microbiota-associated chronic diseases. PloS One 12 (1), e0163956. doi: 10.1371/journal.pone.0163956 28068341 PMC5221766

[B56] Khoruts A. Sadowsky M. J. Hamilton M. J. (2015). Development of fecal microbiota transplantation suitable for mainstream medicine. Clin. Gastroenterol. Hepatol. 13 (2), 246–250. doi: 10.1016/j.cgh.2014.11.014 25460566

[B57] Kimura I. Ozawa K. Inoue D. Imamura T. Kimura K. Maeda T. . (2013). The gut microbiota suppresses insulin-mediated fat accumulation *via* the short-chain fatty acid receptor GPR43. Nat. Commun. 4, 1829. doi: 10.1038/ncomms2852 23652017 PMC3674247

[B58] Kootte R. S. Levin E. Salojarvi J. Smits L. P. Hartstra A. V. Udayappan S. D. . (2017). Improvement of insulin sensitivity after lean donor feces in metabolic syndrome is driven by baseline intestinal microbiota composition. Cell Metab. 26 (4), 611–9.e6. doi: 10.1016/j.cmet.2017.09.008 28978426

[B59] Krauss R. M. (2004). Lipids and lipoproteins in patients with type 2 diabetes. Diabetes Care 27 (6), 1496–1504. doi: 10.2337/diacare.27.6.1496 15161808

[B60] Krischer J. P. Liu X. Vehik K. Akolkar B. Hagopian W. A. Rewers M. J. . (2019). Predicting islet cell autoimmunity and type 1 diabetes: An 8-year TEDDY study progress report. Diabetes Care 42 (6), 1051–1060. doi: 10.2337/dc18-2282 30967432 PMC6609953

[B61] Kumar M. Singh P. Murugesan S. Vetizou M. McCulloch J. Badger J. H. . (2020). Microbiome as an immunological modifier. Methods Mol. Biol. 2055, 595–638. doi: 10.1007/978-1-4939-9773-2_27 31502171 PMC8276114

[B62] Kump P. Wurm P. Grochenig H. P. Wenzl H. Petritsch W. Halwachs B. . (2018). The taxonomic composition of the donor intestinal microbiota is a major factor influencing the efficacy of faecal microbiota transplantation in therapy refractory ulcerative colitis. Aliment Pharmacol. Ther. 47 (1), 67–77. doi: 10.1111/apt.14387 29052237 PMC5765501

[B63] Lee S. J. Rho M. (2022). Multimodal deep learning applied to classify healthy and disease states of human microbiome. Sci. Rep. 12 (1), 824. doi: 10.1038/s41598-022-04773-3 35039534 PMC8763943

[B64] Leiva-Gea I. Sanchez-Alcoholado L. Martin-Tejedor B. Castellano-Castillo D. Moreno-Indias I. Urda-Cardona A. . (2018). Gut microbiota differs in composition and functionality between children with type 1 diabetes and MODY2 and healthy control subjects: A case-control study. Diabetes Care 41 (11), 2385–2395. doi: 10.2337/dc18-0253 30224347

[B65] Liu S. da Cunha A. P. Rezende R. M. Cialic R. Wei Z. Bry L. . (2016). The host shapes the gut microbiota *via* fecal MicroRNA. Cell Host Microbe 19 (1), 32–43. doi: 10.1016/j.chom.2015.12.005 26764595 PMC4847146

[B66] Macfarlane G. T. Macfarlane S. (2011). Fermentation in the human large intestine: its physiologic consequences and the potential contribution of prebiotics. J. Clin. Gastroenterol. 45 Suppl, S120–S127. doi: 10.1097/MCG.0b013e31822fecfe 21992950

[B67] Malmqvist E. Larsson H. E. Jonsson I. Rignell-Hydbom A. Ivarsson S. A. Tinnerberg H. . (2015). Maternal exposure to air pollution and type 1 diabetes–accounting for genetic factors. Environ. Res. 140, 268–274. doi: 10.1016/j.envres.2015.03.024 25880886

[B68] Mobasseri M. Shirmohammadi M. Amiri T. Vahed N. Hosseini Fard H. Ghojazadeh M. (2020). Prevalence and incidence of type 1 diabetes in the world: a systematic review and meta-analysis. Health Promot Perspect. 10 (2), 98–115. doi: 10.34172/hpp.2020.18 32296622 PMC7146037

[B69] Mocanu V. Zhang Z. Deehan E. C. Kao D. H. Hotte N. Karmali S. . (2021). Fecal microbial transplantation and fiber supplementation in patients with severe obesity and metabolic syndrome: a randomized double-blind, placebo-controlled phase 2 trial. Nat. Med. 27 (7), 1272–1279. doi: 10.1038/s41591-021-01399-2 34226737

[B70] Moffa S. Mezza T. Cefalo C. M. A. Cinti F. Impronta F. Sorice G. P. . (2019). The interplay between immune system and microbiota in diabetes. Mediators Inflamm. 2019, 9367404. doi: 10.1155/2019/9367404 32082078 PMC7012204

[B71] Mollayeva T. Thurairajah P. Burton K. Mollayeva S. Shapiro C. M. Colantonio A. (2016). The Pittsburgh sleep quality index as a screening tool for sleep dysfunction in clinical and non-clinical samples: A systematic review and meta-analysis. Sleep Med. Rev. 25, 52–73. doi: 10.1016/j.smrv.2015.01.009 26163057

[B72] Mullaney J. A. Stephens J. E. Costello M. E. Fong C. Geeling B. E. Gavin P. G. . (2018). Type 1 diabetes susceptibility alleles are associated with distinct alterations in the gut microbiota. Microbiome. 6 (1), 35. doi: 10.1186/s40168-018-0417-4 29454391 PMC5816355

[B73] Murri M. Leiva I. Gomez-Zumaquero J. M. Tinahones F. J. Cardona F. Soriguer F. . (2013). Gut microbiota in children with type 1 diabetes differs from that in healthy children: a case-control study. BMC Med. 11, 46. doi: 10.1186/1741-7015-11-46 23433344 PMC3621820

[B74] Nicholson J. K. Holmes E. Kinross J. Burcelin R. Gibson G. Jia W. . (2012). Host-gut microbiota metabolic interactions. Science 336 (6086), 1262–1267. doi: 10.1126/science.1223813 22674330

[B75] Oprita R. Bratu M. Oprita B. Diaconescu B. (2016). Fecal transplantation - the new, inexpensive, safe, and rapidly effective approach in the treatment of gastrointestinal tract diseases. J. Med. Life. 9 (2), 160–162. 27453747 PMC4863507

[B76] Org E. Blum Y. Kasela S. Mehrabian M. Kuusisto J. Kangas A. J. . (2017). Relationships between gut microbiota, plasma metabolites, and metabolic syndrome traits in the METSIM cohort. Genome Biol. 18 (1), 70. doi: 10.1186/s13059-017-1194-2 28407784 PMC5390365

[B77] Ott S. J. Waetzig G. H. Rehman A. Moltzau-Anderson J. Bharti R. Grasis J. A. . (2017). Efficacy of sterile fecal filtrate transfer for treating patients with clostridium difficile infection. Gastroenterology 152 (4), 799–811.e7. doi: 10.1053/j.gastro.2016.11.010 27866880

[B78] Paramsothy S. Kamm M. A. Kaakoush N. O. Walsh A. J. van den Bogaerde J. Samuel D. . (2017a). Multidonor intensive faecal microbiota transplantation for active ulcerative colitis: a randomised placebo-controlled trial. Lancet 389 (10075), 1218–1228. doi: 10.1016/S0140-6736(17)30182-4 28214091

[B79] Paramsothy S. Paramsothy R. Rubin D. T. Kamm M. A. Kaakoush N. O. Mitchell H. M. . (2017b). Faecal microbiota transplantation for inflammatory bowel disease: A systematic review and meta-analysis. J. Crohns Colitis. 11 (10), 1180–1199. doi: 10.1093/ecco-jcc/jjx063 28486648

[B80] Pedersen H. K. Gudmundsdottir V. Nielsen H. B. Hyotylainen T. Nielsen T. Jensen B. A. . (2016). Human gut microbes impact host serum metabolome and insulin sensitivity. Nature 535 (7612), 376–381. doi: 10.1038/nature18646 27409811

[B81] Pitocco D. Di Leo M. Tartaglione L. De Leva F. Petruzziello C. Saviano A. . (2020). The role of gut microbiota in mediating obesity and diabetes mellitus. Eur. Rev. Med. Pharmacol. Sci. 24 (3), 1548–1562. doi: 10.26355/eurrev_202002_20213 32096204

[B82] Qin B. Viera A. J. Muntner P. Plassman B. L. Edwards L. J. Adair L. S. . (2016). Visit-to-Visit variability in blood pressure is related to late-life cognitive decline. Hypertension. 68 (1), 106–113. doi: 10.1161/HYPERTENSIONAHA.116.07494 27217401 PMC4900904

[B83] Que Y. Cao M. He J. Zhang Q. Chen Q. Yan C. . (2021). Gut bacterial characteristics of patients with type 2 diabetes mellitus and the application potential. Front. Immunol. 12, 722206. doi: 10.3389/fimmu.2021.722206 34484230 PMC8415158

[B84] Rapoport E. A. Baig M. Puli S. R. (2022). Adverse events in fecal microbiota transplantation: a systematic review and meta-analysis. Ann. Gastroenterol. 35 (2), 150–163. doi: 10.20524/aog.2022.0695 35479587 PMC8922263

[B85] Raybould H. E. Zumpano D. L. (2021). Microbial metabolites and the vagal afferent pathway in the control of food intake. Physiol. Behav. 240, 113555. doi: 10.1016/j.physbeh.2021.113555 34375620

[B86] Rewers M. Ludvigsson J. (2016). Environmental risk factors for type 1 diabetes. Lancet. 387 (10035), 2340–2348. doi: 10.1016/S0140-6736(16)30507-4 27302273 PMC5571740

[B87] Rinninella E. Raoul P. Cintoni M. Franceschi F. Miggiano G. A. D. Gasbarrini A. . (2019). What is the healthy gut microbiota composition? a changing ecosystem across age, environment, diet, and diseases. Microorganisms 7 (1), 14. doi: 10.3390/microorganisms7010014 30634578 PMC6351938

[B88] Rodriguez-Valera F. Martin-Cuadrado A. B. Rodriguez-Brito B. Pasic L. Thingstad T. F. Rohwer F. . (2009). Explaining microbial population genomics through phage predation. Nat. Rev. Microbiol. 7 (11), 828–836. doi: 10.1038/nrmicro2235 19834481

[B89] Rowland I. Gibson G. Heinken A. Scott K. Swann J. Thiele I. . (2018). Gut microbiota functions: metabolism of nutrients and other food components. Eur. J. Nutr. 57 (1), 1–24. doi: 10.1007/s00394-017-1445-8 PMC5847071 28393285

[B90] Salazar J. Angarita L. Morillo V. Navarro C. Martinez M. S. Chacin M. . (2020). Microbiota and diabetes mellitus: Role of lipid mediators. Nutrients 12 (10), 3039. doi: 10.3390/nu12103039 33023000 PMC7600362

[B91] Samuel B. S. Shaito A. Motoike T. Rey F. E. Backhed F. Manchester J. K. . (2008). Effects of the gut microbiota on host adiposity are modulated by the short-chain fatty-acid binding G protein-coupled receptor, Gpr41. Proc. Natl. Acad. Sci. U S A. 105 (43), 16767–16772. doi: 10.1073/pnas.0808567105 18931303 PMC2569967

[B92] Scheithauer T. P. Dallinga-Thie G. M. de Vos W. M. Nieuwdorp M. van Raalte D. H. (2016). Causality of small and large intestinal microbiota in weight regulation and insulin resistance. Mol. Metab. 5 (9), 759–770. doi: 10.1016/j.molmet.2016.06.002 27617199 PMC5004227

[B93] Segal J. P. Abbasi F. Kanagasundaram C. Hart A. (2018). Does the Internet promote the unregulated use of fecal microbiota transplantation: a potential public health issue? Clin. Exp. Gastroenterol. 11, 179–183. doi: 10.2147/CEG.S159609 29750050 PMC5935081

[B94] Shou J. Chen P. J. Xiao W. H. (2019). The effects of BCAAs on insulin resistance in athletes. J. Nutr. Sci. Vitaminol (Tokyo) 65 (5), 383–389. doi: 10.3177/jnsv.65.383 31666474

[B95] Smillie C. S. Sauk J. Gevers D. Friedman J. Sung J. Youngster I. . (2018). Strain tracking reveals the determinants of bacterial engraftment in the human gut following fecal microbiota transplantation. Cell Host Microbe 23 (2), 229–240.e5. doi: 10.1016/j.chom.2018.01.003 29447696 PMC8318347

[B96] Smits L. P. Kootte R. S. Levin E. Prodan A. Fuentes S. Zoetendal E. G. . (2018). Effect of vegan fecal microbiota transplantation on carnitine- and choline-derived trimethylamine-N-Oxide production and vascular inflammation in patients with metabolic syndrome. J. Am. Heart Assoc. 7 (7), e008342. doi: 10.1161/JAHA.117.008342 29581220 PMC5907601

[B97] Sorbara M. T. Pamer E. G. (2022). Microbiome-based therapeutics. Nat. Rev. Microbiol. 20 (6), 365–380. doi: 10.1038/s41579-021-00667-9 34992261

[B98] Stankov K. Benc D. Draskovic D. (2013). Genetic and epigenetic factors in etiology of diabetes mellitus type 1. Pediatrics 132 (6), 1112–1122. doi: 10.1542/peds.2013-1652 24190679

[B99] Steffes M. W. Sibley S. Jackson M. Thomas W. (2003). Beta-cell function and the development of diabetes-related complications in the diabetes control and complications trial. Diabetes Care 26 (3), 832–836. doi: 10.2337/diacare.26.3.832 12610045

[B100] Stewart C. J. Ajami N. J. O'Brien J. L. Hutchinson D. S. Smith D. P. Wong M. C. . (2018). Temporal development of the gut microbiome in early childhood from the TEDDY study. Nature 562 (7728), 583–588. doi: 10.1038/s41586-018-0617-x 30356187 PMC6415775

[B101] Sun H. Saeedi P. Karuranga S. Pinkepank M. Ogurtsova K. Duncan B. B. . (2022). IDF diabetes atlas: Global, regional and country-level diabetes prevalence estimates for 2021 and projections for 2045. Diabetes Res. Clin. Pract. 183, 109119. doi: 10.1016/j.diabres.2021.109119 34879977 PMC11057359

[B102] Tolhurst G. Heffron H. Lam Y. S. Parker H. E. Habib A. M. Diakogiannaki E. . (2012). Short-chain fatty acids stimulate glucagon-like peptide-1 secretion *via* the G-protein-coupled receptor FFAR2. Diabetes. 61 (2), 364–371. doi: 10.2337/db11-1019 22190648 PMC3266401

[B103] Vatanen T. Kostic A. D. d'Hennezel E. Siljander H. Franzosa E. A. Yassour M. . (2016). Variation in microbiome LPS immunogenicity contributes to autoimmunity in humans. Cell 165 (6), 1551. doi: 10.1016/j.cell.2016.05.056 27259157

[B104] Verbeke F. Janssens Y. Wynendaele E. De Spiegeleer B. (2017). Faecal microbiota transplantation: a regulatory hurdle? BMC Gastroenterol. 17 (1), 128. doi: 10.1186/s12876-017-0687-5 29179687 PMC5704511

[B105] Vermeire S. Joossens M. Verbeke K. Wang J. Machiels K. Sabino J. . (2016). Donor species richness determines faecal microbiota transplantation success in inflammatory bowel disease. J. Crohns Colitis. 10 (4), 387–394. doi: 10.1093/ecco-jcc/jjv203 26519463 PMC4946755

[B106] Vrieze A. Van Nood E. Holleman F. Salojarvi J. Kootte R. S. Bartelsman J. F. . (2012). Transfer of intestinal microbiota from lean donors increases insulin sensitivity in individuals with metabolic syndrome. Gastroenterology. 143 (4), 913–6.e7. doi: 10.1053/j.gastro.2012.06.031 22728514

[B107] Walker D. M. Cates H. M. Loh Y. E. Purushothaman I. Ramakrishnan A. Cahill K. M. . (2018). Cocaine self-administration alters transcriptome-wide responses in the brain's reward circuitry. Biol. Psychiatry 84 (12), 867–880. doi: 10.1016/j.biopsych.2018.04.009 29861096 PMC6202276

[B108] Wang J. Jia H. (2016). Metagenome-wide association studies: fine-mining the microbiome. Nat. Rev. Microbiol. 14 (8), 508–522. doi: 10.1038/nrmicro.2016.83 27396567

[B109] Wang H. Lu Y. Yan Y. Tian S. Zheng D. Leng D. . (2019). Promising treatment for type 2 diabetes: Fecal microbiota transplantation reverses insulin resistance and impaired islets. Front. Cell Infect. Microbiol. 9, 455. doi: 10.3389/fcimb.2019.00455 32010641 PMC6979041

[B110] Wang Z. Xie Z. Lu Q. Chang C. Zhou Z. (2017). Beyond genetics: What causes type 1 diabetes. Clin. Rev. Allergy Immunol. 52 (2), 273–286. doi: 10.1007/s12016-016-8592-1 27878451

[B111] Weingarden A. Gonzalez A. Vazquez-Baeza Y. Weiss S. Humphry G. Berg-Lyons D. . (2015). Dynamic changes in short- and long-term bacterial composition following fecal microbiota transplantation for recurrent clostridium difficile infection. Microbiome 3, 10. doi: 10.1186/s40168-015-0070-0 25825673 PMC4378022

[B112] White P. J. Newgard C. B. (2019). Branched-chain amino acids in disease. Science 363 (6427), 582–583. doi: 10.1126/science.aav0558 30733403 PMC9940269

[B113] Woodworth M. H. Neish E. M. Miller N. S. Dhere T. Burd E. M. Carpentieri C. . (2017). Laboratory testing of donors and stool samples for fecal microbiota transplantation for recurrent clostridium difficile infection. J. Clin. Microbiol. 55 (4), 1002–1010. doi: 10.1128/JCM.02327-16 28077694 PMC5377826

[B114] Zhang F. Zhang T. Zhu H. Borody T. J. (2019). Evolution of fecal microbiota transplantation in methodology and ethical issues. Curr. Opin. Pharmacol. 49, 11–16. doi: 10.1016/j.coph.2019.04.004 31059962

[B115] Zhu C. Song K. Shen Z. Quan Y. Tan B. Luo W. . (2018). Roseburia intestinalis inhibits interleukin17 excretion and promotes regulatory T cells differentiation in colitis. Mol. Med. Rep. 17 (6), 7567–7574. doi: 10.3892/mmr.2018.8833 29620246 PMC5983956

[B116] Ziegler A. G. Nepom G. T. (2010). Prediction and pathogenesis in type 1 diabetes. Immunity. 32 (4), 468–478. doi: 10.1016/j.immuni.2010.03.018 20412757 PMC2861716

[B117] Zuo T. Wong S. H. Lam K. Lui R. Cheung K. Tang W. . (2018). Bacteriophage transfer during faecal microbiota transplantation in clostridium difficile infection is associated with treatment outcome. Gut 67 (4), 634–643. doi: 10.1136/gutjnl-2017-313952 28539351 PMC5868238

